# The isokinetic strength profile of elite soccer players according to playing position

**DOI:** 10.1371/journal.pone.0182177

**Published:** 2017-07-31

**Authors:** Robert Śliwowski, Monika Grygorowicz, Radosław Hojszyk, Łukasz Jadczak

**Affiliations:** 1 Department of Team Sports Games of the University School of Physical Education in Poznań, Poland; 2 Rehasport Clinic FIFA Medical Centre of Excellence, Department of Physiotherapy, Poznań, Poland; Universite de Nantes, FRANCE

## Abstract

The aim of this study was to compare isokinetic strength performance profiles in elite soccer players across different field positions. A total of 111 elite international players of Polish Ekstraklasa (the top division in Poland) were examined during the 2010–2015 seasons. The players were classified into six positional roles: central defenders (CD), external defenders (ED), central midfielders (CM), external midfielders (EM), forwards (F), and goalkeepers (G). The concentric isokinetic strength (peak torque [PT] of quadriceps and hamstrings, H/Q ratios) was calculated for the dominant leg and the non-dominant leg at angular velocity of 1.05 rad ·s^–1^, whereas to assess isokinetic muscle endurance, the total work [TW] at angular velocity of 4.19 rad ·s^–1^, was taken into consideration. The results showed that isokinetic strength performance varies significantly among players in different playing positions. The analysis of PT for quadriceps (PT-Q) and hamstrings (PT-H) generally showed that the goalkeepers and central midfielders had lower strength levels compared to other playing positions. In the case of PT-H and hamstring/quadricep (H/Q) peak torque ratios, statistically significant differences were also noted for the legs, where mean values noted for the dominant leg were higher than for the non-dominant leg. For TW for quadriceps (TW-Q) and hamstrings (TW-H), statistically significant differences were noted only between playing positions. TW-Q values for goalkeepers were lower than for central defenders and external midfielders. TW-H values for goalkeepers were lower than for central midfielders, central defenders and external midfielders. This study showed that specific functional activity of players in individual positions on the field influences the varied profile of isokinetic strength performance.

## Introduction

Measurement of muscle strength is an important factor in the evaluation and prediction of muscular condition in addition to functional capacity [1]. A lot of research supports this concept [2–4]. Isokinetic peak torque (PT) assessment is one of the most commonly applied methods of evaluation of muscle strength of the lower extremities in soccer [5–7]. Knee flexor and extensor muscle strength and the hamstring-to-quadricep ratio have also been identified as important criteria to analyse the risk of lower extremity injuries [2, 6, 8]. Numerous studies have focused on the effect of various training programmes in order to prevent injuries to adult and youth soccer players [9–11]. Several researchers have successfully described the isokinetic muscle profile at various levels of soccer training [12, 13]. Isokinetic muscular strength has also been compared in various sub-periods of the frame [14] or in other sport groups [15].

In the context of a wide area of exploration of isokinetic studies of soccer players, little information is available about the strength isokinetic profile according to the position on the field. Although the existing reports in this area mainly confirm the varied profile of isokinetic strength between players in various positions [16–23], some studies have found contradictory results [16, 17, 21–23]. A study by Tourny-Chollet et al. [20] indicates that forward players essentially showed higher hamstring concentric strengths compared to midfielders and defenders. Öberg et al. [19] noted a significantly higher knee extensor torque in goalkeepers and defenders than in forwards. A greater PT of knee extensor muscles in defensive players compared to midfield players was also confirmed by the recent study of Costa Silva et al. [16]. However, Goulart et al. [17], who used a more advanced division of players, demonstrated that full backs’ knee flexor muscles exhibited lower PT when compared with the other positions, and goalkeepers’ knee extensor muscles exerted lower PT and had a higher fatigue index when compared to results for other positions. These findings are in accordance with Ruas et al. [22], who found that goalkeepers demonstrated different characteristics and concentric PT across muscles than most players in other field positions. However, Magalhães et al. [23] did not note different isokinetic strengths between players of different positions.

These differences between the results are due to a large variability in test protocols, namely speed, type of dynamometer (Biodex, Cybex), large differences in sports level, and the numbers of studied participants, which make the object of the studies ambiguous. A few studies refer to the division of players on the pitch, which has recently been commonly used in the literature, resulting from different functional requirements and motor profile of play in a given position [17, 21, 22]. Some studies relate to young players [16, 17]. Others are not representative enough in terms of the size of the sample (<30) [16, 18, 20]. A prevailing majority of the reports refer their analyses to absolute values of the studied isokinetic indicators [16, 18, 21–23]. In our opinion, significant differences in the build and weight among players in different playing positions [18, 20, 21, 22] may have a significant influence on the level of isokinetic strength. Apart from the study of Goulart et al. [17], no references to total muscle work were found. This variable represents the torque generated during the entire range of motion (ROM) and is related to the muscle energy expenditure during the whole test [24, 25]. Taking this variable into consideration in isokinetic assessment of muscular strength with the use of study protocols with a large number of repetitions may provide important information on muscle endurance [26]. Another limitation of the majority of the studies is the lack of research covering the elite level of sports championship. Performing this type of analysis on top-level soccer players may not only be a valuable cognitive contribution but may also have implications for widely defined team selection and optimising physical and medical preparation strategies for the training process.

This information could stress the need to perform further studies on a larger sample size in order to identify an isokinetic muscle profile in soccer players of the professional elite level, verifying possible adjustments according to game and training specificity. Therefore, the purpose of the current study was to compare isokinetic strength performance profiles (PT and TW of quadriceps and hamstrings, and H/Q ratios) in elite soccer players across different field positions.

## Methods

### Participants, procedures, and data collection

The study covered a group of 111 elite international soccer players of Polish Ekstraklasa (the top division in Poland). The participants (26±5 years; 79±8 kg; 181±7 cm) included players of many nationalities (81 Polish, 13 South European, 5 Latin American, 4 African, 3 Eastern European, 3 Western European, and 2 Northern European). Seventy-eight players (70% of the total) were members of senior and youth national teams of their countries (mainly European countries—92%). The participants had at least three years of experience playing soccer at a professional level with regular training, which constituted a part of their professional contract. The study was performed from 2009 to 2015. All measurements were taken in December, close to the end of the first half of the Ekstraklasa season, 48 hours after the final game.

The players were divided into six subgroups according to playing positions: goalkeepers (n = 14), central defenders (n = 18), external defenders (n = 14), central midfielders (n = 30), external midfielders (n = 14), and forwards (n = 21). The profile of the individual playing positions was based on the different activities on the pitch field and the main area in which this activity was carried out [27–29]. Additionally, for the studies cited above, we decided to enlarge the player groups and include the goalkeeper position as a separate group to be analysed. The players were asked about their playing positions that they had played most frequently in the past year. Only players who played in their usual positions were included in the sample. Moreover, the analysis was conducted on values collected only from players who played at least 50% of official club matches in the season preceding the study. All players in the analysis played in the 1-4-2-3-1 formation. Player and team confidentiality was ensured thanks to anonymity of all performance data. As part of their professional playing contracts, players were informed about the experimental risks and provided written consent for their data to be collected and analyzed. For players younger than 18 years, their parents or guardians were informed of the risks and signed an informed consent before the investigation. The study was carried out according to the Declaration of Helsinki, and the protocol was fully approved by the Bioethical Committee at the Poznań University of Medical Sciences. The basic physical characteristics of the players are presented in Table 1.

**Table 1:** Physical characteristics and training experience of the subjects (mean values ± SD).

	G	CD	ED	CM	EM	F
	(n = 14)	(n = 18)	(n = 14)	(n = 30)	(n = 14)	(n = 21)
**Body height** **[cm]**	190.77^#^ ±4.324	187.12^##^ ±4.511	181.21^ ±2.694	180.17^^ ±5.133	176.57 ±3.756	184.38^###^ ±5.352
**Body mass** **[kg]**	88.08^$^ ±6.873	81.08^$ $^ ±6.097	75.93 ±3.075	75.31 ±5.200	71.71 ±5.369	79.19^$ $ $^ ±6.523
**Age** **[years]**	25.75 ±4.539	25.12 ±4.566	25.29 ±4.375	26.61 ±6.039	24.54 ±4.051	24.79 ±4.404
**Training experience** **[years]**	15.23 ±2.257	16.14 ±2.568	16.23 ±2.137	16.83 ±3.167	16.32 ±2.335	16.46 ±2.428

^#^G > EM^c^, CM^c^, ED^c^, F^c^, CD^a^;

^##^CD > EM^c^, CM^c^, ED^c^;

^###^F > EM^c^, CM^a^, ED^a^;

^ED > EM^a^;

^^CM > EM^a^;

^$^G > EM^c^, CM^c^, ED^c^, F^b^, CD^a^;

^$ $^CD > EM^c^, ED^a^, CM^b^;

^$ $ $^F > EM^c^

Significantly higher value of the body height and mass across playing position: P < 0.05^a^, P < 0.01^b^, P < 0.001^c^

### Test procedures (muscle strength profiling)

The measurements were performed by the examiner team in the laboratory for isokinetic testing at the Rehasport Clinic FIFA Medical Centre of Excellence in Poznań, Poland. In this study, the measurement of isokinetic knee muscle strength (as measured by peak torque [PT]) and muscle endurance (as measured by total work [TW]) was performed using the Biodex System 3 (Biodex Medical Systems^™^ Inc., New York, USA) dynamometer. According to many authors [24, 30] both variables (PT and TW) express strength performance well.

The proper positioning and stabilisation of the subject, the alignment between the axis of the rotation of the machine and the knee joint, and the gravity compensation and the elimination of acceleration artefacts were performed according to the instruction manual for the Biodex Medical Systems and were similar to the ones described in the literature [1, 7, 10]. The warm-up that each player performed before the isokinetic assessment took 10–15 minutes and consisted of mild pedalling on a stationary Monark cycle ergometer at a moderate pace (50–100 W) and dynamic stretches for the major lower-limb muscle groups [1, 5]. The concentric isokinetic torque of the quadriceps and hamstrings was assessed during continuous (bidirectional) knee extension–flexion movements at the angular velocity of 1.047 rad ·s^-1^ and 4.189 rad ·s^-1^ through a knee range of motion of 0° (flexed) to 90° (full extension). The above testing speeds have been widely used in other studies assessing muscle strength in soccer players [2, 7, 13]. Participants were given three trials at sub-maximal efforts with a gradually increasing load (50%, 75%, and approximately 100% of maximum capability) and then performed one set of three repetitions at the maximal concentric contraction at angular velocities of 1.047 rad ·s^-1^ and, subsequently, 30 repetitions at angular velocities of 4.189 rad ·s^-1^. Then, the same protocol was followed with the opposite leg. The participants were requested to resist the powered leg extension movement as hard as they could. A 30-s rest was given after the third sub-maximal trial, a one-minute break was given between two angular velocities, and a three-minute break was given when the machine setting was changed for the opposite leg. It has been reported that this number of repetitions has a high reliability for isokinetic testing. Standardised verbal encouragement was given before each maximal effort, and visual feedback of the recorded torque was provided. The order of testing was randomised for the dominant (D) and non-dominant (N-D) legs [6, 31]. Limb dominance was defined as the leg that is preferred when kicking a ball, and this was determined through an interview [1, 10]. In order to limit the analysis to constant velocity periods, only windowed data has been used. The statistical analysis included the relative PTs (N·m·kg^-1^) for flexors (PT-Q) and extensors (PT-H) in both legs and the unilateral ratio of muscle torque for both the dominant and non-dominant extremities (H_D_/Q_D_ and H_N_/Q_N_, respectively) at angular velocity of 1.05 rad ·s^–1^ and, similarly, the relative TWs (J ·kg^-1^) for flexors (TW-Q) and extensors (TW-H) for both legs at angular velocity of 4.19 rad ·s^–1^. Previous studies [32] indicated that TW measured during a protocol involving 30 reciprocal maximal concentric contractions represented a good compromise between reliability (intraclass coefficient of correlation = 0.91 and standard error of measurement = 4%) and bioenergetical interpretability of the data.

All tests took place between 9:00 am and 1:00 pm and were conducted in the same order for each player. The same member of the research team performed all isokinetic tests. The evaluated players were not exposed to intensive training for two days before testing. Before the start of the study, the subjects were asked to fill in a questionnaire to determine whether they had any musculoskeletal pain, discomfort, or known injury in a lower extremity. Subjects were free of previous significant knee injuries and had no history of ACL repairs or rehabilitation. Furthermore, the participants had no history of a lower extremity fracture or surgery during the year prior to the study. Those players who reported a major or moderate lower extremity injury or any injury to the knee or thigh were excluded from further analysis.

### Statistical analyses

All statistical analyses were conducted using STATISTICA 10.0 for Windows Version (SPSS Inc., Chicago, IL, USA). All results are reported as means and standard deviations (mean SD) calculated by conventional procedures, unless otherwise stated. The normality of the variables was tested with the Shapiro–Wilk Test, and the coefficients of asymmetry and kurtosis were found. For all isokinetic variables (PT and TW of the quadriceps and hamstrings per body mass, and H/Q ratios), differences between legs (dominant, non-dominant) were tested using the T-test. Differences between positions on the field (goalkeepers, central midfielders, external defenders, central midfielders, external midfielders, and forwards) were tested using one-way analysis of variance (ANOVA). Duncan post-hoc procedures were used to identify specific differences. In the absence of interactions, only main effects were analysed. This analysis was the basis for creating models of multifactorial analysis of variance (ANOVA) for each isokinetic variable (ANOVA) when significant interactions were present. A 2x6 (leg x position) repeated-measures ANOVA was used to compare only PT of the hamstrings. Statistical significance was set at an alpha of 0.05 for all statistical procedures.

## Results

The physical characteristics of the players demonstrated significant differences in their height and weight according to their playing positions (see Table 1). Goalkeepers are significantly heavier than players in all other positions (in all cases *p* < 0.0001, except for central defenders *p* = 0.0006). Central defenders are significantly heavier than external midfielders (*p* < 0.0001), external defenders (*p* = 0.0128), and central midfielders (*p* = 0.0065). On the other hand, forwards are significantly heavier than external midfielders (*p* = 0.0004).

**Fig 1:**
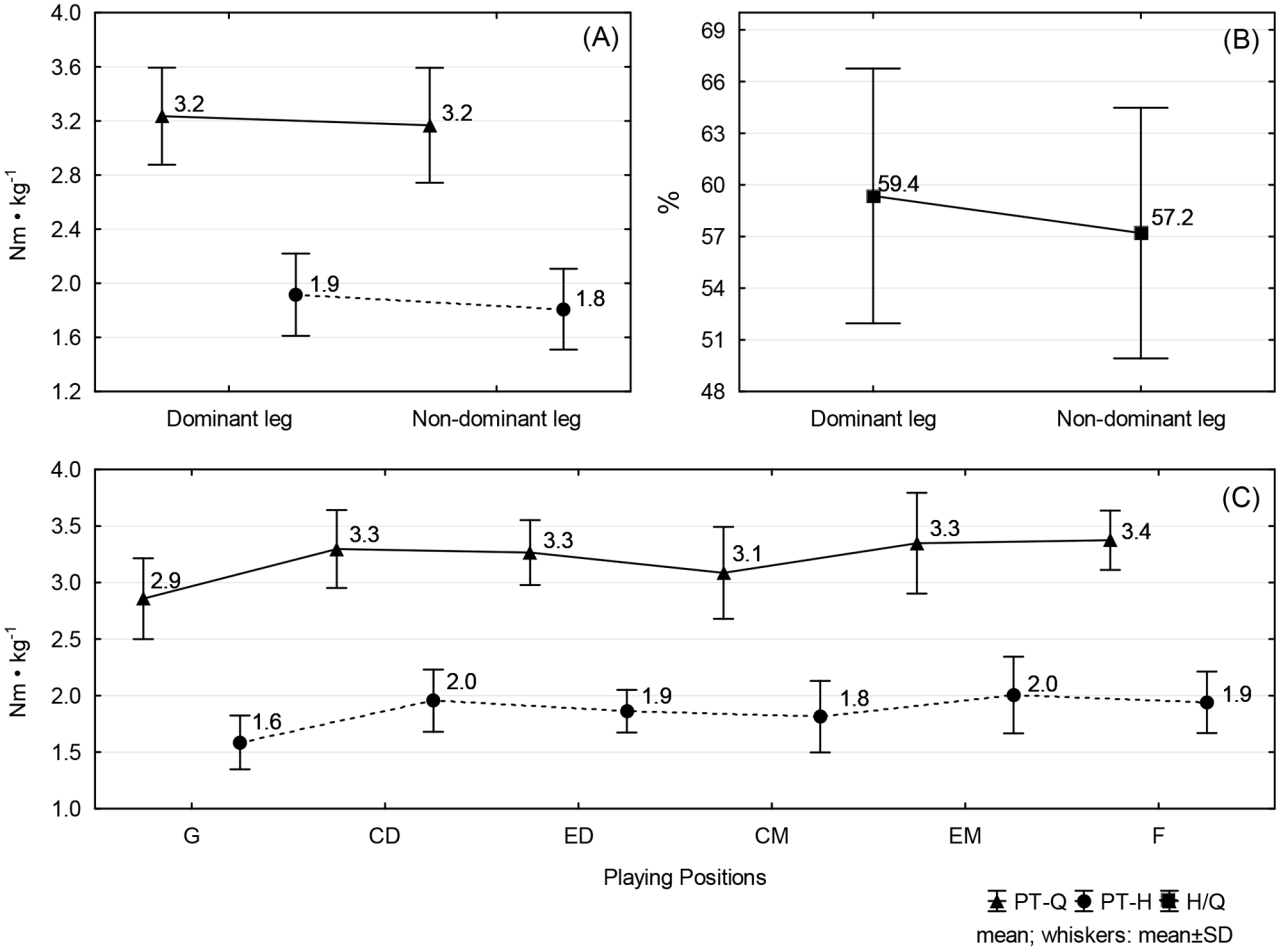
Mean values and standard deviation for: (A) PT/BM of the quadriceps and hamstrings by legs (dominant, non-dominant); (B) H/Q ratios by legs (dominant, non-dominant); (C) PT/BM of the quadriceps and hamstrings by playing positions (G, CD, ED, CM, EM, F).

Fig 1 presents the mean values of PT-Q and PT-H for legs and playing positions and mean values of H/Q ratios for legs. For legs, statistically significant differences were noted only for PT-H and H/Q ratios (*p* = 0.0109 and *p* = 0.0320, respectively; Fig 1A and 1B), where mean values noted for the dominant leg were higher than for the non-dominant leg. For PT-Q and PT-H, statistically significant differences were also noted between playing positions (in both cases *p* < 0.0001; Fig 1C). PT-Q values for goalkeepers were lower than for external defenders, central defenders, external midfielders, and forwards, whereas for central midfielders they were lower than for external midfielders, and forwards. PT-H values for goalkeepers were lower than for central midfielders, external defenders, central defenders, forwards, and external midfielders, whereas for central midfielders they were lower than for central defenders and external midfielders.

**Fig 2:**
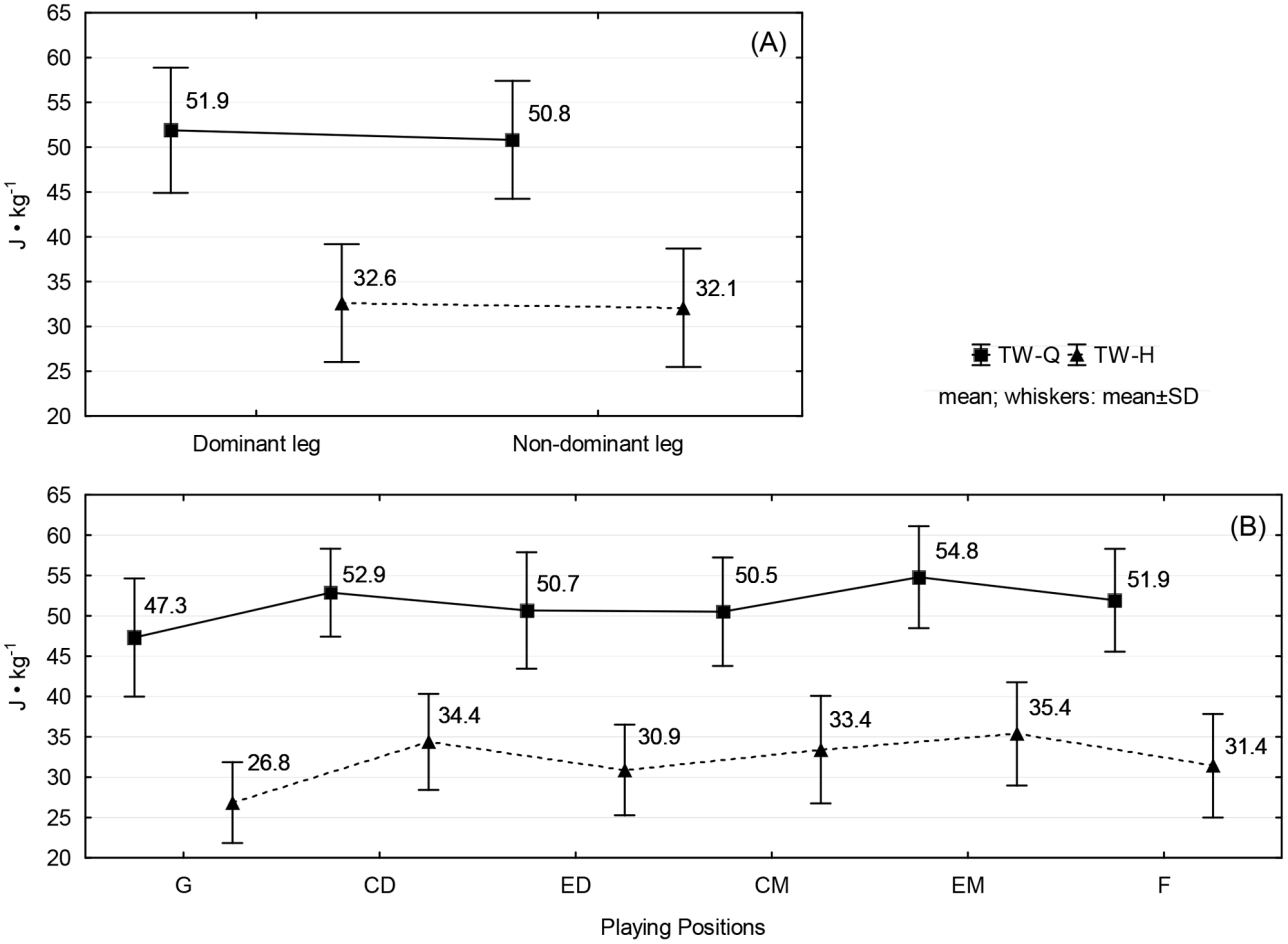
Mean values and standard deviation for TW/BM of the quadriceps and hamstrings by: (A) legs (dominant, non-dominant); (B) playing positions (G, CD, ED, CM, EM, F).

Fig 2 presents the mean values of TW-Q and TW-H for legs and playing positions. For TW-Q and TW-H, statistically significant differences were noted only between playing positions (in both cases *p* < 0.0001). TW-Q values for goalkeepers were lower than for central defenders and external midfielders. TW-H values for goalkeepers were lower for central midfielders, central defenders and external midfielders.

For PT-H, there were no interactions. However, there was a main effect for position (*p* < 0.0001), in which PT-H values for goalkeepers were lower than for central midfielders, external defenders, forwards, central defenders, and external midfielders, whereas for central midfielders they were lower than for central defenders and external midfielders, and for legs (*p* = 0.0055), where mean values noted for the dominant leg were higher than for the non-dominant leg (Table 2).

**Table 2:** Characteristics the PT/BM of the quadriceps and hamstrings, and H/Q ratios at 1.05 rad ·s^–1^ angular velocity across playing positions (mean ± SD).

Variables	Leg	G^a^ (n = 14)	CD (n = 18)	ED (n = 14)	CM^b^ (n = 30)	EM (n = 14)	F (n = 21)
**PT-Q**	**Dominant**	2.91 ±0.276	3.29 ±0.271	3.32 ±0.266	3.1 ±0.401	3.33 ±0.412	3.47 ±0.223
	**Non-dominant**	2.81 ±0.455	3.31 ±0.415	3.21 ±0.301	3.07 ±0.446	3.37 ±0.491	3.28 ±0.271
**PT-H**	**Dominant**	1.66 ±0.222	2.02 ±0.261	1.83 ±0.186	1.85 ±0.347	2.11 ±0.292	2.02 ±0.275
	**Non-dominant**	1.51 ±0.253	1.89 ±0.281	1.89 ±0.193	1.79 ±0.308	1.91 ±0.363	1.86 ±0.247
**H/Q**	**Dominant**	57.42 ±7.224	60.61 ±7.937	54.99 ±4.686	61.05 ±7.672	62.21 ±8.469	58.23 ±6.273
	**Non-dominant**	56.08 ±7.499	57.32 ±8.712	59.29 ±3.252	57.49 ±6.546	56.17 ±10.038	56.69 ±7.051

^a^Indicates a significantly greater PT-H then CM (p = 0.0006), ED (p < 0.0001), F (p < 0.0001), CD (p < 0.0001), and EM (p < 0.0001)

^b^Indicates a significantly greater PT-H then CD (p = 0.0493), and EM (p = 0.0087)

## Discussion

The various demands of elite soccer isokinetic strength have been frequently described in the scientific literature. However, few studies attempted to address specific profiles of isokinetic strength in reference to the playing positions. In the context of recent highly advanced playing position specialisation and, what follows, a varied motor profile in a given playing position, these aspects are of increased significance. Therefore, the aim of this study was to compare isokinetic strength performance profiles in elite soccer players across different field positions.

The principal finding of the present study was that concentric isokinetic strength varies significantly among players in different playing positions. In terms of the PT for quadriceps, goalkeepers had lower values than central midfielders, external defenders, central defenders, external midfielders, and forwards, whereas the values were lower for central midfielders than for external midfielders, and forwards (*p* < 0.0001; Table 2). The latest study of Costa Silva et al. [16], referenced at the beginning, indicates that statistically significant differences among players in the three specified playing positions (defenders, midfielders, forwards) related only to the PT of extensor muscles, where defenders had statistically higher values than midfielders but showed no difference compared to forwards. According to the authors, these results can be justified due to the performance of similar specific actions between defenders and forwards, mainly composed of short and intense movements like sprints and jumps, demanding great effort of knee extensors. Contrary to these findings, Öberg et al. [19], based on a similar division of players, found that goalkeepers and defenders had greater quadricep strength compared to forwards. These findings are in accordance with Ruas et al. [22], who found that goalkeepers’ quadricep strength was greater compared with all other positions (side backs, central defender midfielders, central attacking midfielders, and forwards), and central backs had greater quadricep strength compared to central defender midfielders. However, Carvalho and Cabri [21] demonstrated that goalkeepers and forwards have greater quadricep strength in the dominant leg, while centre backs have greater quadricep non-dominant leg strength compared with all other positions. Contrary to these findings, there are reports that do not show statistically significant differences in the level of concentric PT for quadriceps between players in different positions [16–18, 20, 23].

In soccer practice, it is normally understood that the quadricep muscle plays an important role in the execution of sprints, jumps, kicks, and passes, while the hamstrings mainly act as stabilisers of the knee joint during changes of speed and direction and kicking [18, 21, 22, 33, 34]. In sprinting, turns and tackling of the hamstrings are used concentrically but are mainly used eccentrically to control, decelerate, and stabilise the knee [2, 7, 9, 22, 33, 34]. The results found in this study were similar for quadriceps, where goalkeepers had lower PT of the hamstrings relative to all other positions, and central midfielders relative to central defenders and external midfielders (*p* < 0.0001; Table 2). These findings are similar to the data obtained by Tourny-Chollet et al. [20], who found that midfielders have lower concentric hamstring strength in the non-dominant leg compared to forwards and defenders, and for the dominant leg compared to forwards alone (goalkeepers were not considered in this study). Weber et al. [18], based on the same division of players, demonstrated the same relations, where defenders had higher values for flexor strength muscles compared to midfielders for the dominant leg (no statistically significant differences were noted for the non-dominant leg). On the other hand, Goulart et al. [17] showed higher average PT of right knee flexors in central defenders compared to central midfielders, external defenders, and forwards. Similar relations were noted by Carvalho and Cabri [21], who noted that players in almost all field positions had lower hamstring strength for both dominant and non-dominant legs than goalkeepers and central backs.

Briefly, we found that the goalkeepers and central midfielders had lower strength levels of extensors and flexors compared to other field positions. The results of goalkeepers may be explained by their specific tasks, which may have contributed to these results. Furthermore, Ruas et al. [22] suggest that the results of the goalkeepers should be interpreted differently than other positions. Contrary to the studies referred to above [21, 22], our goalkeepers had the lowest levels of PT of the quadriceps and hamstrings for both legs (Table 2). Such contradictory findings should be considered in the context of the lack of relativisation of analysed isokinetic indicators to players’ weight. It is generally known in the literature that goalkeepers have higher weight compared to other players [35]. On the other hand, the differences noted between field players can be justified by the performance of similar specific actions among central and external defenders, external midfielders, and forwards, mainly composed of short and intense movements, like sprints and jumps, demanding great effort of the knee extensors compared to central midfielders, whose activities display characteristics of a more prolonged action [16]. Consequently, the general trend confirmed by many studies [16, 18, 20, 21, 23] is that central midfielders have a lower level of quadricep and hamstring muscle strength compared to players in other positions on the field.

According to Fousekis at al. [3], certain skills in soccer such as kicking, passing, and cutting are distinctly unilateral, require asymmetrical motor patterns, and lead to the development of more force on one side toward the other. This is in agreement with the results of many earlier reports [36, 37], which showed statistically significant differences in muscle strength between the dominant leg and the non-dominant leg. On the contrary, our study does not confirm these differences with respect to extensor muscles among players in different positions. The lack of significant differences in PT values for the quadriceps between the dominant leg vs. non-dominant leg is similar to the results noted by Rahnama et al. [5], Costa Silva et al. [16], Weber et al. [18] and Magalhães et al. [23], who also did not record significant differences between the dominant and non-dominant strength of the players in different field positions. This result may have been influenced by the methodology used in the training of both limbs to perform technical movements [16], as well as by the versatility of tasks performed, which has led us in recent years to eliminate differences in the muscle force profile of the players in different positions [21]. In contrast to the extensors, for flexors we noted statistically significant differences between legs, where mean values for the dominant leg were higher than for the non-dominant leg (Table 2). These findings, in turn, are in accordance with the results of studies using both concentric [18, 20, 21] and eccentric contraction strength flexors [2, 9, 18, 22]. Perhaps in both cases there is a similar pattern of adaptive changes.

These data did not show statistically significant differences in the case of H/Q ratios among different field positions (Table 2). As in our study, Costa Silva et al. [16], Weber et al. [18], and Ruas et al. [22] did not report statistically significant differences between groups in relation to conventional H/Q ratio for the dominant and non-dominant legs. The lack of any variation between groups in the case of the analysed indicator shows that it should be mainly of diagnostic significance in the assessment of injury risk in players. It seems, however, that PT ratio measurements have greater importance for clinical evaluation than performance in soccer [4]. Although the present study found significant differences in H/Q ratios between legs, where mean values noted for the dominant leg were higher than for the non-dominant leg, these results indicated a better stability of the knee joint for the dominant leg. On the other hand, these findings concerning conventional H/Q ratios were not confirmed in the studies referred to above [16, 18, 22]. We also found the values of conventional H/Q ratio ranging from 55% to 62% at 1.05 rad ·s^–1^. The above values are similar to those reported by other authors [3, 13, 21, 22, 33]. Previously, the H/Q ratios of less than 60% for the angular velocity at 1.05 rad ·s^–1^ were linked to ACL injury; knee joint stabilization is affected by activation of the quadriceps and relative weakness of the hamstrings [14, 15, 22, 38–40]. Therefore, particularly important factors in monitoring the effects of training programmes and in injury prevention are the evaluation and control of muscle strength [2].

Although PT is considered as the favoured measure of isokinetic strength performance, from the practical point of view other measures such as TW may provide significant information in terms of the muscle ability to generate strength over a longer time by players in specific positions. It might seem that due to significantly different functional requirements in the game, players in different positions on the pitch will represent various levels of muscle endurance. Our study shows that TW-Q values for goalkeepers were lower than for central defenders and external midfielders, and TW-H values for goalkeepers were lower than for central midfielders, central defenders and external midfielders (Table 3). Although the highest mean values of TW-Q and TW-H were clearly noted for external midfielders and central defenders, these differences between individual outfield players did not show statistical significance (a tendency to statistical significance between external midfielders and external defenders for TW-H should only be noted here). These findings are similar to the data obtained by Goulart et al. [17], who also did not report statistically significant differences in TW values between players in specific positions on the pitch. According to the authors [17], this trend may be due to the fact that muscle endurance training in clubs is similar for all players, which may explain the lack of differences in this respect between players in specific positions on the pitch. In our opinion in the context of modern training technologies, these loads should be strongly individualised in terms of individual components of strength performance.

**Table 3:** Characteristic the TW/BM of the quadriceps and hamstrings at 4.19 rad ·s^–1^ angular velocity across playing positions (mean ± SD).

Variables	Leg	G (n = 14)	CD (n = 18)	ED (n = 14)	CM (n = 30)	EM (n = 14)	F (n = 21)
**TW-Q**	**Dominant**	48.38 ±6.363	53.45 ±5.574	51.78 ±7.351	50.22 ±7.321	55.16 ±6.904	52.99 ±6.939
	**Non-dominant**	46.25 ±8.287	52.29 ±5.424	49.54 ±7.171	50.79 ±6.188	54.41 ±5.919	50.87 ±5.728
**TW-H**	**Dominant**	27.19 ±5.423	34.41 ±6.069	31.15 ±5.248	33.37 ±6.721	35.88 ±6.536	32.09 ±6.591
	**Non-dominant**	26.49 ±4.758	34.35 ±6.012	30.64 ±6.146	33.45 ±6.728	34.85 ±6.456	30.75 ±6.326

Although the relationships between isokinetic muscular strength in soccer players and functional demands in play are not clear, it has been suggested that the specific functional activity of players in individual positions in the field, may influence isokinetic strength profile [18, 20, 21]. The present results confirmed these relations especially in terms of muscle strength, where the goalkeepers and central midfielders had lower strength levels of extensors and flexors compared to other field positions. The results for muscle endurance looked slightly different, as the goalkeepers had lower results compared to external midfielders and central defenders. Goalkeepers, however, due to the specificity of play in their position and a significantly lower level of these elements, had the lowest values of most analysed isokinetic indicators. Therefore, it can be assumed that that specific functional activity of players in individual positions in the field influences the varied profile of isokinetic strength performance. This study may be a key part of the trend of activities aiming to individualise the training process in soccer and the preparation strategies in terms of strength preparation of players in individual positions and in the context of profiling players for a given position. However, future reports should also take into consideration deficiencies and imbalances in strength and endurance for individual muscle groups between players of different positions in the field.
